# Task shifting in Mozambique: cross-sectional evaluation of non-physician clinicians' performance in HIV/AIDS care

**DOI:** 10.1186/1478-4491-8-23

**Published:** 2010-10-12

**Authors:** Paula E Brentlinger, Américo Assan, Florindo Mudender, Annette E Ghee, José Vallejo Torres, Pilar Martínez Martínez, Oliver Bacon, Rui Bastos, Rolanda Manuel, Lucy Ramirez Li, Catherine McKinney, Lisa J Nelson

**Affiliations:** 1 International Training and Education Center on HIV, Department of Global Health, School of Public Health , University of Washington, Seattle, Washington, USA; 2 Direcção Nacional de Assistência Médica, Ministry of Health, Maputo, Mozambique; 3 International Training and Education Center on HIV, Maputo, Mozambique; 4 International Training and Education Center on HIV, University of California, San Francisco, California, USA; 5 Centers for Disease Control and Prevention, Global AIDS Program, Maputo, Mozambique

## Abstract

**Background:**

Many resource-constrained countries now train non-physician clinicians in HIV/AIDS care, a strategy known as 'task-shifting.' There is as yet no evidence-based international standard for training these cadres. In 2007, the Mozambican Ministry of Health (MOH) conducted a nationwide evaluation of the quality of care delivered by non-physician clinicians (*técnicos de medicina*, or TMs), after a two-week in-service training course emphasizing antiretroviral therapy (ART).

**Methods:**

Forty-four randomly selected TMs were directly observed by expert clinicians as they cared for HIV-infected patients in their usual worksites. Observed clinical performance was compared to national norms as taught in the course.

**Results:**

In 127 directly observed patient encounters, TMs assigned the correct WHO clinical stage in 37.6%, and correctly managed co-trimoxazole prophylaxis in 71.6% and ART in 75.5% (adjusted estimates). Correct management of all 5 main aspects of patient care (staging, co-trimoxazole, ART, opportunistic infections, and adverse drug reactions) was observed in 10.6% of encounters.

The observed clinical errors were heterogeneous. Common errors included assignment of clinical stage before completing the relevant patient evaluation, and initiation or continuation of co-trimoxazole or ART without indications or when contraindicated.

**Conclusions:**

In Mozambique, the in-service ART training was suspended. MOH subsequently revised the TMs' scope of work in HIV/AIDS care, defined new clinical guidelines, and initiated a nationwide re-training and clinical mentoring program for these health professionals. Further research is required to define clinically effective methods of health-worker training to support HIV/AIDS care in Mozambique and similarly resource-constrained environments.

## Background

In sub-Saharan Africa, the human immunodeficiency virus (HIV) epidemic has expanded rapidly, access to antiretroviral therapy (ART) is often inadequate, and shortages of health workers and infrastructure are often critical [1]. Task-shifting, defined as "a process of delegation of tasks to health workers with lower qualifications," is one strategy for increasing the availability of HIV/AIDS treatment in such environments [2,3]. The World Health Organization (WHO) recently called for more research to define standardized methods for health worker training and to assure quality of care in support of task-shifting [3].

In 2004, when the Mozambican Ministry of Health (MOH) first contemplated task-shifting, Mozambique estimated that nationwide adult HIV seroprevalence was 16.2%, 1.5 million citizens were infected with HIV, and the health workforce included only 662 physicians (0.35/10 000 population) and 2698 non-physician clinicians (1.43/10 000 population) [4,5]. Mozambique's strategic plan for 2004-2008 mandated expansion of ART from 17 to 129 health units, with the aim of increasing enrolled patients from 7924 to 132 280 nationwide, and increasing ART availability in rural and peri-urban areas [5]. Task-shifting and decentralization were to be supported by rapid training and deployment of non-physician clinicians known as '*técnicos de medicina' *(TMs).

Because pre-service training for TMs (30 months' duration, after completion of the 10^th ^grade) did not yet include HIV/AIDS content, MOH developed a new in-service training course. Its curriculum emphasized ART and co-trimoxazole prophylaxis, with lesser attention given to clinical staging of HIV/AIDS [6] and to opportunistic infections (OIs). This reflected the plan's intent that TMs only care for stable, uncomplicated, ambulatory non-pregnant adults in WHO clinical stages I and II [6], and that they not initiate ART, although they were authorized to provide follow-up care for stable patients on first-line antiretrovirals that had been prescribed by physicians. The two-week duration was the norm for in-service training in Mozambique. The course was taught to TMs in all 10 provinces in 2006 and 2007. During this period, policy was changed to authorize TMs to initiate first-line ART without physician consultation. Shortly after the deployment of the first graduates, MOH received anecdotal reports of deficiencies in quality of care, and decided to conduct a nationwide evaluation of quality of HIV/AIDS-related care as provided by TMs.

### Objectives

The aim of the study was to develop a Mozambique-specific evidence base to guide improvements in training of TMs. The primary objectives were to describe the extent to which TMs correctly identified the WHO clinical stage of HIV-infected patients, managed co-trimoxazole prophylaxis and ART, and diagnosed and managed adverse drug reactions (ADRs) and opportunistic infections (OIs). Secondary objectives included qualitative descriptions of the TMs' clinical environment (e.g. human and material resource availability), and of health workers' attitudes toward HIV/AIDS training.

## Methods

### Study design

This cross-sectional evaluation used direct observation of the clinical practice of randomly selected TMs, supplemented by semi-structured interviews with key informants. Two trained clinical observers (COs) observed each TM as he or she cared for HIV-infected ambulatory adults (> = 18 years) (See Additional File 1 for a description of the selection and training of the COs). For each TM, we attempted to observe 3 patient encounters: 1 first visit of a newly diagnosed patient, 1 scheduled follow-up visit, and 1 unscheduled urgent visit. Without interrupting the consultation, the COs recorded the findings of each medical history and physical exam as conducted by the TM, using standardized instruments. After the TM completed his or her evaluation, but before the TM discharged the patient, the COs asked the TM to report the patient's diagnoses and WHO clinical stage, and the proposed plan for management of co-trimoxazole, ART, OIs, and ADRs. This conversation took place in private, out of earshot of the patient. If it was necessary to confirm the TM's clinical findings, the COs then repeated some or all of the history, physical examination, or chart review (also recording their own findings on standardized instruments). COs and TMs then finalized the patient care plan in another private discussion, and the TM communicated the final plan to the patient. The COs were careful not to express any criticism or disagreement with the TM in the presence of the patient. On the same day, the COs conducted semi-structured interviews with the TMs, their clinical supervisors, and health unit administrators. The interviews focused on TM demographic information, perceived strengths and weaknesses of the ART course, and the health unit's human and material resources.

### Setting

Data collection occurred in Mozambican public-sector health facilities in which the TM participants normally practiced, in all 10 provinces and in Maputo, the capital city, from October through December 2007.

### Participants

Mozambican TMs who had completed the 2-week in-service course and were actively managing ART in the public sector comprised the study population.

MOH and non-governmental organization training lists, as verified by provincial HIV/AIDS coordinators, were used to construct a sampling frame (described in Additional File 2). TMs located more than 4 hours' drive from provincial capitals were excluded for logistical reasons.

TMs who had served as in-service course instructors, or on the study team, were also ineligible. Because very few TMs provided ART-related services without having attended the in-service course, no untrained comparison group was available.

Two urban and 2 rural TMs were randomly selected in each province, and 4 urban TMs in Maputo City. Only 1 TM was selected per health unit. Administrators and clinical supervisors assigned to the selected health units served as key informants.

The first eligible patients presenting during each observation session were asked to participate; random sampling of patients was not feasible.

### Variables

Correct clinical management was defined as directly observed clinical performance that conformed to Mozambican standards as taught in the in-service course in the opinions of both the COs and the study team leader, who reviewed all completed clinical observation instruments with the COs [7].

Correct clinical staging was defined as identification of WHO clinical stage in conformity with year 2004 WHO criteria (in effect when the ART course was designed) [8] or the year 2006 WHO criteria (partially disseminated at the time of the study) [6]. Because staging required identification of OIs, resource constraints influenced interpretation of this standard. TMs were given credit for correct performance if they made appropriate use of available diagnostic studies, and were not penalized for not initiating evaluations that were not feasible in their environments.

Correct management of co-trimoxazole was defined as initiation or continuation of co-trimoxazole prophylaxis in patients with confirmed HIV infection, WHO clinical stages 3 or 4 or CD4+ T-lymphocyte (CD4) count <200 cells/mm^3^, and no known allergy to sulfonamides; or discontinuation of co-trimoxazole in a patient who had had an adverse drug reaction (ADR) or whose CD4 count was > = 200 cells/mm^3 ^on the 2 most recent measurements.

Correct management of ART was defined as initiation or continuation of the first-line regimen (stavudine [d4T], lamivudine [3TC], and nevirapine [NVP]) in a patient with confirmed HIV infection, WHO clinical stage 4 or CD4 <200 cells/mm^3^, and no contra-indications to first-line therapy (e.g. pregnancy, active tuberculosis (TB), ADR, laboratory abnormalities, or unstabilized OI); or discontinuation or modification of ART in a patient who had experienced a significant ADR or developed another contra-indication to first-line therapy.

Because Mozambican national norms for co-trimoxazole and ART initiation were modified between the development of the in-service course and the initiation of the study (the CD4 threshold for initiating co-trimoxazole increased to 350 cells/mm^3^, and ART eligibility criteria were expanded to include patients in clinical stage 3 with CD4 < 350 cells/mm3), TMs whose clinical decisions conformed to the new but not to the old norms were also given credit for correct performance.

### Data sources

The clinical observation instruments were adapted from those used to evaluate Mozambique's Integrated Management of Childhood Illness (IMCI) program [9,10] and a Mozambican OI training course [11], and recorded findings of the medical history, physical exam, laboratory results, and the *técnicos*' clinical management decisions for each observed patient (the instrument is reproduced in Additional file 3).

Descriptions of TM demographic and professional characteristics and of health unit characteristics were based on semi-structured interviews with key informants.

### Bias

We attempted to minimize observer bias by using 2 COs for each patient observation. In each 2-observer team, there was no more than 1 clinician who had served as an instructor in the ART course for TMs, and COs were not permitted to evaluate TMs whom they supervised directly. We also used each patient's clinical data as recorded on the observation instrument to validate COs' conclusions. For example, we reviewed the patient's clinical stage, medication allergies, and CD4 count in order to confirm that the TM's management of co-trimoxazole was consistent with Mozambican norms, the patient's clinical presentation, and the CO's assessment. We attempted to minimize sampling bias by constructing the TM sampling frame from multiple, frequently updated sources.

### Sampling

For this descriptive study, we sought a randomized sample of 44 TMs and their respective health units. This was estimated to be a 21% sample of all MOH health units with ART capacity. We estimated that a clinical observation team could visit a maximum of 4 health units in a single week of field work. We did not prepare a formal power calculation because no prior data described the frequency of clinical errors in this setting. Random-number tables were used to guide selection of TMs from each province's list of trained, practicing TMs.

### Statistical methods

Quantitative analyses described the proportion of patient encounters in which each primary clinical domain (staging, co-trimoxazole, ART, ADRs, or OIs) was managed correctly by the TM, as recorded by COs. Sampling weights (calculated separately for each province) were used to reflect the probability of selection of each TM, and robust confidence intervals were calculated to reflect likely correlation of results for patients attended by the same TM [12]. It was not possible to adjust for the total number of HIV-infected patients under each TM's care, because patients were not assigned to specific clinicians' panels.

We also constructed a dichotomized composite variable representing TM and CO concordance in all 3 of the following domains: clinical staging, co-trimoxazole management, and ART management. Using logistic regression, we calculated odds ratios (ORs) and their 95% confidence intervals (CIs) for association of TM, health unit, and patient characteristics and this indicator of correct diagnosis and management in both bivariate and multivariate analyses. To account for correlation between patients seen by the same TM, we used the robust sandwich estimate of standard error around odds ratios reported.

### Ethical considerations

The study protocol was approved by the Mozambican National Bioethics Committee. Secondary data analysis was authorized by the University of Washington Human Subjects Division. This study also underwent review at the Centers for Disease Control and Prevention, where it was deemed to be a non-research program evaluation. Participating MOH staff (TMs, clinic managers and clinical supervisors) gave written informed consent. Patients gave oral consent. TM and patient data were confidential.

Clinical mentoring by the COs was incorporated into the protocol based on the ethical principle of beneficence, and allowed patients to benefit directly from clinical observer correction of clinical omissions or mistakes. Immediate clinical feedback was also of benefit to the TMs themselves.

## Results

### Description of the sample

Six hundred and sixty-nine *técnicos *were initially reported to have received ART training. After inspection of training lists and consultation with provincial HIV/AIDS coordinators, 53% of these names were discarded (25% duplicates, 21% not active in HIV/AIDS clinical care, 1% not trained in ART, 6% other reasons). We were often unable to ascertain the status of TMs stationed in remote rural districts.

No eligible and available TM refused to participate. The characteristics of the observed TMs and the health units at which they practiced are described in Tables 1 and 2.

**Table 1:** Description of participating *técnicos de medicina* (n = 44)

Characteristic (n = 44)	N (%)	Median (Range)	Missing
Age (years)		30 (23-64)	1

Female	14 (32.6%)		1

Years since completion of pre-service training		5.5 (0.5-24.5)	2

Months since completion of in-service ART training		13 (3-24)	2

Also completed in-service course on opportunistic infections	24 (54.6%)		0

Months of experience providing ART		14 (4-80)	2

Number of patients on ART seen in health unit during month prior to study		145 (9-2090)	4

Number of patients started on ART by TM during preceding month		12 (3-60)	5

Notes:

ART: antiretroviral therapy

TM: *técnico de medicina*

**Table 2:** Description of participating health units (n = 44)

Characteristic	N (%)	Median (range)	Missing
Urban	22 (50.0%)		0

Months since health unit introduced ART		21 (1-71)	5

Number of ART-related patient encounters during most recent month		145 (9-2090)	4

TM's tasks include:			

Staging	37 (88.1%)		2

Initiate co-trimoxazole	38 (90.5%)		2

Request CD4 count	36 (87.8%)		3

Request other labs	39 (95.1%)		3

Diagnose and treat OI	40 (97.6%)		3

Initiate ART	38 (90.5%)		2

In-hospital care for patients on ART	18 (46.2%)		5

Laboratory and imaging capacity (on-site availability of test)			

CD4 count	6 (14.6%)		3

Complete blood count	30 (75.0%)		4

Transaminases	19 (48.7%)		5

Hemoglobin	37 (88.1%)		2

Sputum smear microscopy for detection of acid-fast bacilli	41 (97.6%)		2

CSF cell count	20 (55.6%)		8

CSF india ink preparation	15 (39.5%)		6

Rapid malaria test	42 (97.7%)		1

Chest radiograph	14 (35.0%)		4

Notes:

ART: antiretroviral therapy

CD4 count: CD4+ T-lymphocyte count

CSF: cerebrospinal fluid

OI: opportunistic infection

TM: *técnico de medicina*

We observed 127 patient consultations (3 per TM, except in 5 health units with low caseloads). The characteristics of the observed patients are given in Table 3.

**Table 3:** Description of participating patients (n = 127)

Patient characteristics	N	%	Median (range)	Missing
Age (years)			33 (19-62)	2

Female	82	65.6%		2

Pregnant	8	6.3%		0

Type of visit				12

New patient	37	32.2%		

Follow-up	60	52.2%		

Urgent care	18	15.7%		

Most recent CD4+ T-lymphocyte count (cells/mm^3^)*				40

<200	30	34.5%		

200-349	25	28.7%		

> = 350	32	36.8%		

On ART	58	45.7%		0

ART Regimen (any)**				

NVP+AZT (antenatal regimen)	1	0.8%		

3TC+NVP+AZT	7	5.5%		

3TC+NVP+d4T	48	37.8%		

3TC+EFV+d4T	2	1.6%		

Active tuberculosis (TB)***	11	8.7%		0

TB treatment status				0

Newly diagnosed - not yet treated	2	1.6%		

Intensive phase	3	2.4%		

Continuation phase	5	4.0%		

Treatment interruption	1	0.8%		

Taking co-trimoxazole prophylaxis	46	36.2%		0

Notes: * Twenty-five of the patients who had no CD4+ T-lymphocyte result recorded were new patients.

** ART: antiretroviral therapy. AZT: zidovudine. d4T: stavudine. EFV: efavirenz. NVP: nevirapine. 3TC: lamivudine.

*** Positive sputum smear for acid-fast bacilli recorded during observed patient encounter, and/or patient receiving TB treatment through national TB control program.

### Main results

We observed a broad range of clinical practice quality. Table 4 describes concordance between clinical observers and TMs in 5 major domains: staging (37.6% agreement), co-trimoxazole management (71.6% agreement), ART management (75.5% agreement), ADR management (69.7% agreement), and diagnosis of OIs and other infectious diseases (49.1% agreement). In 89.4% of observed encounters, the COs disagreed with the TMs about diagnosis or management in one or more of these domains. In all cases, the level of agreement was significantly different from 100%.

**Table 4:** Main outcomes: agreement between clinical observers and *técnicos de medicina* (n = 127)

Domain	**Percentage of 127 patient encounters in which COs and TMs agreed, with 95% C.I**.	
	**Crude estimate**	Adjusted estimate*

Determination of WHO clinical stage of HIV-related illness	37.0 (28.5, 45.5)	37.6 (27.0, 48.2)

Management of co-trimoxazole prophylaxis	72.4 (64.6, 80.3)	71.6 (60.6, 82.6)

Management of antiretroviral therapy	78.0 (70.6, 85.3)	75.5 (66.0, 85.0)

Diagnosis and management of adverse drug reactions	72.3 (63.4, 81.2)	69.7 (57.3, 82.0)

Diagnosis and management of opportunistic and other infections	53.2 (44.3, 62.0)	49.1 (35.4, 62.9)

Agreement on clinical stage, co-trimoxazole prophylaxis, antiretroviral therapy, adverse drug reactions, *and *opportunistic or other infection	12.6 (6.7, 18.4)	10.6 (3.7, 17.6)

Notes: *Estimates adjusted for sampling probability and for clustering among patients seen by the same *técnico de medicina*.

### Staging and opportunistic infections

Differences of opinion between COs and TMs were of three main types: Understaging (miscategorizing a patient as having less advanced disease), overstaging (miscategorizing a patient as having more advanced disease), and premature staging (assigning a clinical stage before completing the indicated clinical and laboratory evaluations). Correct staging is predicated on correct identification of OIs, which was difficult in this resource-constrained setting. In particular, Stage IV OIs could be diagnosed only infrequently. Examples of staging difficulties are described in Additional file 4.

### Co-trimoxazole prophylaxis, antiretroviral therapy

Disagreements were of 2 main types: initiation or continuation of medications when not indicated, and failure to initiate or continue when co-trimoxazole and/or ART were indicated. Examples are given in Additional files 5 and 6.

### Adverse drug reactions

ADRs were confirmed by the CO in 20 patients (15.7%), and were often under-diagnosed or under-treated by the TMs. Examples are given in Additional file 7.

### Correlates of correct management

Bivariate and multivariate analyses revealed three principal correlates of correct patient management (defined as concordance in the three principal domains of staging, co-trimoxazole management, and ART management). In multivariate analyses, increasing ART caseloads at the TM's home health unit were positively correlated with correct performance in all 3 domains (OR per additional patient on ART per month 1.001 [95% CI 1.000, 1.002]), while increasing TM age and the presence of any sign or symptom of TB or of confirmed TB were both negatively associated with correct performance (OR per year of TM age 0.896 [95% CI 0.831, 0.965]; OR if patient had confirmed or suspected TB 0.132 [95% CI 0.019, 0.936]). No other observed characteristic of patients, TMs, or health units was significantly associated with 'correct' performance.

## Discussion

### Key results

We found that, in the majority of observed patient encounters, Mozambican non-physician clinicians who had received brief in-service HIV/AIDS training did not adhere to Mozambican national clinical standards as taught in their course. These assessments were based on direct observation of patient care by experienced clinicians familiar with the Mozambican clinical environment. Although some errors were unlikely to have had adverse effects on patient outcomes, others were more serious, and even life-threatening. However, we also observed TMs who provided excellent patient care.

Better TM performance was correlated with younger TM age, the absence of confirmed TB or TB symptoms in the observed patients, and higher ART caseloads at the TMs' home health facilities. The younger TMs may have performed better because they began their pre-service training after pre-service faculty had begun to acquire expertise of their own in HIV/AIDS care. Worse TM performance when faced with TB patients or TB suspects may be the result of Mozambican policy restricting TB/HIV co-infection care to physicians; or may be a marker for worse performance in the presence of symptomatic patients in general. Better performance in health facilities with larger numbers of patients on ART may reflect the impact of better-evolved systems for support of HIV/AIDS care. However, because our data were cross-sectional and our patient numbers small, we are not able to draw firm conclusions from the observed associations.

To the best of our knowledge, this study is the first to use direct observation of patient care to describe the quality of HIV/AIDS care in the context of task-shifting in a highly resource-constrained environment, using a randomized national sample of providers.

### Limitations

Because we were unable to follow patients longitudinally, we cannot link observed clinical practice to patient outcomes. However, the process indicators we examined are significantly linked to patient outcomes in multiple published studies [13-16].

The cross-sectional design and the paucity of laboratory and imaging support also resulted in a high proportion of encounters in which available patient data were insufficient to justify specific diagnostic, staging, and/or management decisions. However, had we conducted the study in a better-resourced environment, the results would not have reflected actual clinical practice in the TMs' worksites.

Because we did not use standardized patients [17], some differences in observed clinical performance can be ascribed to differences in the complexity of the observed patients.

We were not able to describe the TMs' level of exposure to other HIV/AIDS training (in addition to the ART course). For example, we were unable to obtain data on the quantity or quality of post-course hours of expert clinical mentoring, or provincial-level variations in ART course design and implementation. Some of the observed variation in clinical performance may have been caused by these unmeasured differences. Our subjective impression was that the most proficient TMs had served long apprenticeships with expert HIV/AIDS clinicians.

Exclusion of TMs based in the most remote rural sites may have resulted in overestimation of the quality of HIV/AIDS care as delivered by TMs, because medications, medical equipment, and clinical supervisors were all less likely to reach these health units. If so, this constraint to generalizability actually strengthens our conclusions.

## Conclusions

Faced with a widespread HIV/AIDS epidemic and extreme constraints in human and material resources, Mozambique shifted day-to-day responsibility for HIV/AIDS-related patient care, including care of critically ill patients, from physicians to non-physician clinicians. Our findings suggest that Mozambique's 2-week in-service ART training strategy did not result in creation of adequate TM capacity to provide high-quality clinical HIV/AIDS services.

There are two likely explanations - not mutually exclusive - for these findings. First, the content and methods of the 2-week in-service course were not adequate to provide the TMs with the skills and knowledge needed to attend a high-volume, symptomatic, and challenging patient population. Course duration was almost certainly too short, and many assumptions that drove curriculum development (TMs would see only stable, non-pregnant adult patients in clinical stages 1 or 2; clinical staging was feasible in this environment; TMs would not initiate ART; TMs would be supervised by physicians) proved to be incorrect. Course content had significant lacunae; for example, the TMs were not taught standard guidelines for differential diagnosis, diagnosis and management of most OIs, and they were not taught to diagnose or manage most severe ADRs.

Second, newly trained TMs were often deployed to health units that lacked both clinical mentoring and material resources; these constraints often prohibited both the use and further development of the TMs' knowledge and skills. The excellent clinical practice that we observed on some occasions suggests that properly supported, more experienced TMs are able to perform at a substantially higher clinical level than what was observed in the overall sample.

Although there is only sparse published literature on health worker performance following HIV/AIDS training, the quality of care difficulties that we observed are consistent with those described after the introduction of other health initiatives [18-20]. Particularly important is the precedent set by the IMCI strategy. Although the IMCI interventions are substantially less complex than HIV/AIDS care, program evaluations have consistently shown that brief in-service trainings alone do not result in adequate adherence to clinical guidelines, and that training must be augmented by construction of context-specific, evidence-based guidelines, drug-delivery systems, post-training clinical supervision and other health-systems support [21-24]. Indeed, the IMCI strategy's use of direct clinical observation of IMCI trainees' clinical practice, as conducted by a silent observer using a standardized clinical checklist, was one of our primary methodological inspirations.

However, evaluations of other programs have demonstrated that the combination of health worker training and focused, sustained systems support can indeed result in substantial improvements in patient outcomes [25,26].

### Generalizability

These findings were not intended to be generalizable to other cadres of health workers (e.g. physicians or nurses), or to settings other than the Mozambican public sector. However, the synergistic problems of high HIV seroprevalence, extreme resource constraints, and ineffectual health worker training in HIV/AIDS treatment are not unique to Mozambique, and other programs may find these results relevant [1,27,28]. The study was not intended to evaluate the broader strategies of task-shifting and decentralization.

### Policy implications and Mozambique's response to the study findings

Immediately upon dissemination of preliminary study findings, MOH recognized that the study results had important implications for Mozambican policy, particularly with regard to health-worker training and the definition and promulgation of cadre-specific standards for clinical care. In consequence, MOH suspended the in-service ART training, re-evaluated the TMs' HIV/AIDS-related scope of work, and began drafting new guidelines for diagnosis and management of OIs and other complications of AIDS and AIDS treatment. In November 2008, MOH initiated a new program to re-train TMs in HIV/AIDS care; this program includes both didactic sessions and long-term, workplace-based clinical mentoring. The new interventions will also undergo rigorous evaluation, in order to create a Mozambique-specific evidence base to support future approaches to clinically effective health-worker training and health policy. Finally, MOH is currently integrating HIV/AIDS content into the pre-service curriculum for TMs to ensure that these providers possess the necessary competencies when they enter the workforce.

## Abbreviations used

The following abbreviations, listed in alphabetical order, were used in this paper: ADR: adverse drug reaction; AIDS: acquired immune deficiency syndrome; ART: antiretroviral therapy; AZT: zidovudine; CD4: CD4+ T-lymphocyte; CI: confidence interval; CSF: cerebrospinal fluid; CO: clinical observer; d4T: stavudine; EFV: efavirenz; HIV: human immunodeficiency virus; IMCI: Integrated Management of Childhood Illness; MOH: Mozambican Ministry of Health; NVP: nevirapine; OI: opportunistic infection; OR: odds ratio; TB: tuberculosis; TMs: *técnicos de medicina *(Mozambican mid-level non-physician clinicians); WHO: World Health Organization; 3TC: lamivudine.

## Competing interests

Three of this paper's co-authors (LRL, CM, LJN) serve as technical advisors to the funder in Mozambique. The authors have no other conflicts of interest or competing interests to declare.

## Authors' contributions

AA, PEB, CM, LJN, and LRL designed the study. PEB, AEG, PMM, and JVT participated in both data collection and data coding; OB also participated in data collection. PEB and AEG conducted the data analysis. All authors participated in data interpretation. PEB drafted this paper; all authors participated in critical revision of the manuscript, and all approved the final version.

## Supplementary Material

Additional file 1 **Selection and training of clinical observers**. Click here for file

Additional file 2 **Sampling frame for *técnicos de medicina***. Click here for file

Additional file 3 **The clinical observation instrument**. Click here for file

Additional file 4 **Clinical staging and opportunistic infection diagnosis: examples of concordance and disagreement between clinical observers and *técnicos de medicina***. Click here for file

Additional file 5 **Co-trimoxazole prophylaxis: examples of concordance and disagreement between clinical observers and *técnicos de medicina***. Click here for file

Additional file 6 **Antiretroviral therapy: examples of concordance and disagreement between clinical observers and *técnicos de medicina***. Click here for file

Additional file 7 **Adverse drug reactions: examples of concordance and disagreement between clinical observers and *técnicos de medicina***. Click here for file
