# Supplementary material for: Task shifting in Mozambique: cross-sectional evaluation of non-physician clinicians' performance in HIV/AIDS care
Source: Hum Resour Health. 2010 Oct 12;8:23. doi: 10.1186/1478-4491-8-23 (PMC2994547; doi:10.1186/1478-4491-8-23)
Supplement: Additional file 1 — Selection and training of clinical observers. [file 1478-4491-8-23-S1.DOC]

## Additional file 1 - Selection and training of clinical observers

The Mozambican Ministry of Health’s national HIV/AIDS program asked provincial HIV/AIDS coordinators to suggest experienced HIV/AIDS clinicians for consideration as clinical observers (COs). Specifically, the Ministry requested the names of physicians and *técnicos de medicina* (TMs) who had cared for HIV-infected patients on ART in Mozambican public-sector facilities and who had served as instructors in the in-service training course or as supervisors of TMs trained in the course. Clinicians who had served as course trainers were specifically sought because of their familiarity with ART course material and the TM scope of practice. The national-level HIV/AIDS program reviewed each potential observer’s qualifications in consultation with the provincial HIV/AIDS coordinators and representatives of other programs engaged in HIV/AIDS care in the relevant provinces before finalizing the selection. The clinicians ultimately selected to participate as clinical observers included physicians and TMs whose primary clinical assignments were in HIV/AIDS treatment programs operated by the Mozambican Ministry of Health, the Mozambican Armed Forces, and non-governmental organizations who collaborated with the Ministry of Health (e.g. Doctors without Borders (*Médicos Sem Fronteiras*; Belgian and Swiss missions), Health Alliance International (*Aliança Internacional para a Saúde*; University of Washington), International Center for AIDS Care and Treatment Programs (ICAP; Columbia University), and the University of Ghent).

Because these expert clinicians could not be spared from their usual worksites for long periods, the COs were scheduled to rotate weekly. Before initiating field work in each province, the study team trained new COs using a standardized protocol and a handbook describing standard study procedures. The study plan and instruments were presented. The study team then enacted standardized clinical scenarios (one member of the study team played the role of a patient, the other the role of a TM), and the clinical observers practiced using the clinical observation tool. At the end of the standardized scenario, the study team reviewed the clinical observers’ practice instruments.

This initial training was reinforced during the actual field work. The COs nominated by the provinces were paired with clinician members of the study team, who also served as clinical observers throughout the six-week course of data collection. Each CO pair thus included one clinician nominated by the provincial health departments (usually a clinician who had participated in ART training for the TMs), and one clinician from the study team; study team members had not served as trainers of the observed TMs and were not affiliated with the provincial health departments. The clinician members of the study team reviewed the other observers’ completed study instruments at the end of each clinical observation session, in order to confirm the completeness and accuracy of the observation record.
