# Supplementary material for: Task shifting in Mozambique: cross-sectional evaluation of non-physician clinicians' performance in HIV/AIDS care
Source: Hum Resour Health. 2010 Oct 12;8:23. doi: 10.1186/1478-4491-8-23 (PMC2994547; doi:10.1186/1478-4491-8-23)
Supplement: Additional file 2 — Sampling frame for técnicos de medicina. [file 1478-4491-8-23-S2.DOC]

## Additional file 2 - Sampling frame for *técnicos de medicina*:

One year prior to the initiation of data collection, the study team gathered lists of *técnicos de medicina* (TMs) trained in the 2-week in-service course from the Mozambican Ministry of Health (MOH) at the national level, and from non-governmental organizations active in health worker training at the provincial level. Three months prior to data collection, the study team requested updated training lists from the above-mentioned sources and from the provincial health departments. We sought TM training lists from multiple sources in order to minimize the likelihood that our sampling frame would exclude TMs who had been trained but whose names had not been officially reported to MOH. All lists contained the trained TMs’ names and last known health unit assignment. The lists were consolidated and sorted by province. Lists were reviewed to detect and eliminate duplicates based on both name and health unit while preserving partial mismatches. Random numbers were then assigned to the remaining TMs. Immediately prior to the initiation of field work, the study team confirmed the names, worksites, and availability of the apparently eligible TMs with the provincial-level HIV/AIDS coordinator, beginning with the lowest random number and continuing until 4 eligible and available TMs (2 urban and 2 rural in each province; 4 urban in the City of Maputo) had been identified as study participants.
