# Supplementary material for: Task shifting in Mozambique: cross-sectional evaluation of non-physician clinicians' performance in HIV/AIDS care
Source: Hum Resour Health. 2010 Oct 12;8:23. doi: 10.1186/1478-4491-8-23 (PMC2994547; doi:10.1186/1478-4491-8-23)
Supplement: Additional file 3 — The clinical observation instrument. [file 1478-4491-8-23-S3.DOC]

## Additional file 3 - The clinical observation instrument

The clinical observation instrument was designed to fit the full history and physical examination on two sides of a single sheet of A4 paper, so that observers would not have to manage multiple pages during patient encounters. An English translation of the instrument is included below. No personal identifiers of either *técnicos de medicina* (TMs) or patients were recorded; each study instrument was assigned a unique identification number.

During their observation of medical history-taking, clinical observers (COs) checked “Y” if the TM asked the relevant question and the patient answered ”yes”, “N” if the TM asked the question and the patient answered ”no”, “NS” if the TM asked but the patient was unable or unwilling to answer, and “NP” if the TM did not inquire.

During their observation of the physical exam, observers checked the corresponding box if the TM reported that any abnormal finding was present, or the “normal” (or equivalent) box if the TM conducted the relevant exam but did not report any abnormalities. The observers checked the “NF” box if the TM did not conduct the relevant examination.

The COs used black ink to record the TMs’ performance and clinical conclusions. When the observers conducted their own, confirmatory evaluations later in the visit, they used red ink – on the same copy of the clinical observation instrument – to record discrepancies between (a) the TMs’ clinical evaluation and conclusions and (b) their own.

The observers used additional pages to record other comments and concerns as needed.

**Cover Page of Instrument**

Date: (d)______/(m)_______/2007 Observer(s): __  __  __ __ __

Language of patient (other than Portuguese): ________ Translation?  Yes  No

Informed consent obtained from patient (oral) and from the *técnico*?  Yes  No

Gender of patient:  F  M Age of patient: _______ years old

**Page 1 of Instrument**

| Visit? 1st 2nd 3rd 4th 5th >5 | | Is result of HIV test confirmed positive?  Yes  No | | |
| --- | --- | --- | --- | --- |
| **Medication:**  Previous ART?  Y  N  NP  NS If YES: *Regimen:*  NVP 1 dose  3TC+NVP+AZT   3TC+NVP+D4T  NP  NS  Other (specify) _____________________________________   TB active ( intensive phase  continuation phase  only isoniazid  NP )  CTX preventive  Other ___________________________________________________ | | | | |
| **Allergy to Medication**:   None  CTX  SP  Other __________________________________________  NP  NS | | | | |
| **Previous history**:   Previous TB  Previous OI  Other _­­­­­­­­­­­­­­­­­_______________________________________  NP  NS | | | | |
| **If female, now pregnant**:  Yes  No LMP: ____/____/_____  NP  NS | | | | |
| **Current symptoms** (Reported by nurse or other health worker, before consultation? ⁪ Yes No) | | | | |
| Fever: |  Y  N  NP  NS | | Nausea: |  Y  N  NP  NS |
| Night sweats: |  Y  N  NP  NS | | Vomiting: |  Y  N  NP  NS |
| Weight loss:  Specify % _____ |  Y  N  NP  NS | | Pain or difficulty on swallowing: |  Y  N  NP  NS |
| Burning, tingling, loss or change of sensation: |  Y  N  NP  NS | | Diarrhea:  >1 week?  Y  N  Blood?  Y  N |  Y  N  NP  NS |
| Able to work: |  Y  N  NP  NS | | Abdominal pain: |  Y  N  NP  NS |
| Bedridden:  % of the time: _____ |  Y  N  NP  NS | | Itching: |  Y  N  NP  NS |
| Coughing:  > 3 weeks:  Y  N |  Y  N  NP  NS | | Rash: |  Y  N  NP  NS |
| Chest pain: |  Y  N  NP  NS | | Other skin lesions: |  Y  N  NP  NS |
| Shortness of breath: |  Y  N  NP  NS | | Genital problem: |  Y  N  NP  NS |
| Mouth problems |  Y  N  NP  NS | | Myalgias: |  Y  N  NP  NS |
| Loss of appetite: |  Y  N  NP  NS | | Convulsions: |  Y  N  NP  NS |
| Headache: |  Y  N  NP  NS | | Anxiety: |  Y  N  NP  NS |
| Depression: |  Y  N  NP  NS | | Other: ________________________ | |

| **Physical examination**: Temperature _____C/F Weight ______ kg Height ________ m | | |
| --- | --- | --- |
| General: |  alert  lethargic  wasting  jaundice  agitation   other______________________________________ |  NF |
| Skin: |  normal  vesicles  pustules  scaling  nodules  papules  Kaposi   lymphadenopathy  abscess  erythema  pus  wound  ecchymosis   other ______________________________________ |  NF |
| Mouth: |  normal  candida  gingivitis  abscess  Kaposi   other_______________________________________ |  NF |
| Lungs: |  clear  rhonchi  crackles  dyspnea  wheezes  diminished  RR ___________  retractions  other ___________________________ |  NF |
| Cardio-  vascular: |  BP_______  pulse_________  murmur  gallop  rub   other________________________________________ |  NF |
| Abdomen: |  benign  tenderness  distension  organomegal*y* (liver? spleen?)   abnormal sounds  ascites  other _____________________________ |  NF |
| Genitalia: |  benign  discharge  ulcers  other _______________________ |  NF |
| Neuro: |  benign  meningismus  paresthesia  focal deficit   Disorientation, confusion  other_____________________________ |  NF |

**Page 2 of Instrument**

| Laboratory or Imaging studies with available results reviewed by *técnico*:   Hemogram  CD4  VL  Transaminases  Amylase  Cholesterol  Triglycerides  Glucose   BUN  Creatinine  RPR  HepB  HepC  Malaria (RT?)  Pregnancy  AFB  CXR Other__________________ CD4 Result? ___________ Date of last CD4 ____/____/_____  Describe abnormal results: ____________________________________________________________ |
| --- |

**Page 3 of Instrument**

| WHO disease stage:  I  II  III  IV (Same as it appears on patient chart?  Y  N) | | |
| --- | --- | --- |
| Eligible for CTX:  Y  N | Eligible for ART:  Y  N | Prepared for ART:  Y  N |
| OIs:  Y  N Specify: _________________________________________________________  Adverse reactions to medication?  Y  N Specify: _________________________________  Other problems:  Y  N Specify _______________________________________________ | | |

| PLAN:  CTX:  Start  Stop  Continue  Mosquito Net:  Recommend  Provide  Refer:  Where ____________  OI Tx: *Specify:_____________________________*  Radiology:  CXR  Other_____________ | ART:  NVP  3TC  D4T30  D4T40   EFV  AZT   Start  Stop  Continue  Refer  Laboratory tests: ______________________  Tx Other conditions (e.g. malnutrition):  *Specify* ________________________________  Date of next visit: ____/_____/________ |
| --- | --- |
| Comments: | |
