# Supplementary material for: Task shifting in Mozambique: cross-sectional evaluation of non-physician clinicians' performance in HIV/AIDS care
Source: Hum Resour Health. 2010 Oct 12;8:23. doi: 10.1186/1478-4491-8-23 (PMC2994547; doi:10.1186/1478-4491-8-23)
Supplement: Additional file 4 — Clinical staging and opportunistic infection diagnosis: examples of concordance and disagreement between clinical observers and técnicos de medicina. [file 1478-4491-8-23-S4.DOC]

Additional file 4 - Clinical staging and opportunistic infection diagnosis: examples of concordance and disagreement between clinical observers and *técnicos de medicina*.

| **Cases in which the clinical observer and TM agreed on clinical stage (37.0% of 127 patient encounters)** | |
| --- | --- |
|  | Disseminated Kaposi’s sarcoma, correctly staged as IV. |
|  | Pott’s disease, correctly staged as IV. |
|  | Recent pulmonary TB, correctly staged as III. |
|  | History of varicella zoster, correctly staged as II. |
|  | Asymptomatic, correctly staged as I. |
| **Cases in which the clinical observer believed that the stage was higher than the stage assigned by the TM (15.0%)** | |
|  | Patient receiving treatment for extrapulmonary TB: the TM failed to elicit this history. |
|  | Patient with disseminated Kaposi’s sarcoma: the TM did not examine the patient’s skin lesions and did not detect this condition. |
|  | Patient with pleural TB: the TM did not recognize that the patient’s lung exam was abnormal. |
|  | Patient with severe bacterial infection of skin and underlying muscle (or bone): the TM did not recognize the association between severe soft-tissue infections and HIV staging. |
|  | Patient who had recently had pulmonary TB, successfully treated: the TM down-staged from III to I, because the patient felt better. |
| **Cases in which the clinical observer believed that the stage was lower than that assigned by the TM (16.5%).** | |
|  | The TM assigned stage III for “persistent vulvovaginal candidiasis” in a woman who had had only 1 episode of vaginitis that responded promptly to syndromic treatment. |
|  | The TM assigned stage III for “persistent fever” in an afebrile patient who had had a recent episode of malaria that responded promptly to 1^st^ line malaria treatment. |
|  | The TM assigned stage II for a positive rapid plasma reagin test. |
|  | The TM assigned stage III based on pulmonary TB – but the clinical observer found that the TB had occurred 21 years earlier, presumably before the onset of HIV infection. |
| **Cases in which the TM assigned a clinical stage, but the clinical observer felt that more information was needed before staging (31.5%).** | |
|  | Patient(s) with fever, cough, weight loss, and/or night sweats, diagnostic testing for tuberculosis not yet initiated, staged as III for “pulmonary TB.” |
|  | Patient with diarrhoea and the subjective report of weight loss, not yet treated with antibiotics, no stool exams available in this setting, staged as III. |
|  | Patient with severe anaemia, cause not yet determined, too early to judge response to presumptive treatment with antimalarials and ferrous sulfate, staged as III. |
|  | Asymptomatic patients who had been started on ART in other health units (sometimes in other countries) and arrived for follow-up without medical records. |
