# Supplementary material for: Task shifting in Mozambique: cross-sectional evaluation of non-physician clinicians' performance in HIV/AIDS care
Source: Hum Resour Health. 2010 Oct 12;8:23. doi: 10.1186/1478-4491-8-23 (PMC2994547; doi:10.1186/1478-4491-8-23)
Supplement: Additional file 5 — Co-trimoxazole prophylaxis: examples of concordance and disagreement between clinical observers and técnicos de medicina. [file 1478-4491-8-23-S5.DOC]

## Additional file 5 - Co-trimoxazole prophylaxis: examples of concordance and disagreement between clinical observers and *técnicos de medicina*

| **Cases in which the clinical observer and the TM agreed on co-trimoxazole management (72.4% of 127 encounters)** | |
| --- | --- |
|  | Patient with Kaposi’s sarcoma and CD4 count < 200. TM correctly continued co-trimoxazole. |
|  | New patient, asymptomatic, no CD4 count available yet. TM correctly decided not to start co-trimoxazole. |
| **Cases in which the TM believed that co-trimoxazole was indicated but the clinical observer disagreed (12.6%).** | |
|  | Patient whose previous 2 CD4 counts were over 500 cells/mm^3^. TM did not realize that co-trimoxazole could be discontinued. |
|  | New patient, asymptomatic, no CD4 yet. The local norm (not consistent with Mozambican national norms) was to start all patients on co-trimoxazole prophylaxis at the first visit, then discontinue it if the first CD4 was >= 500 cells/mm^3^. |
|  | Patient with previous allergic reaction to co-trimoxazole; TM had not inquired about drug allergies. |
| **Cases in which the TM believed that the patient did not require co-trimoxazole but the clinical observer disagreed (15.0%).** | |
|  | Patient initiating ART; TM believed that it was necessary to discontinue co-trimoxazole when ART was initiated. |
|  | Symptomatic patient with CD4 count of 49 cells/mm^3^. |
