# Supplementary material for: Task shifting in Mozambique: cross-sectional evaluation of non-physician clinicians' performance in HIV/AIDS care
Source: Hum Resour Health. 2010 Oct 12;8:23. doi: 10.1186/1478-4491-8-23 (PMC2994547; doi:10.1186/1478-4491-8-23)
Supplement: Additional file 6 — Antiretroviral therapy: examples of concordance and disagreement between clinical observers and técnicos de medicina. [file 1478-4491-8-23-S6.DOC]

## Additional file 6 - Antiretroviral therapy: examples of concordance and disagreement between clinical observers and *técnicos de medicina*

| **Cases in which the clinical observer and the TM agreed on ART management (78.0% of 127 patient encounters).** | |
| --- | --- |
|  | Patient in clinical stage I with CD4 count of 752 cells/mm^3^; TM decided that ART was not indicated. |
|  | Patient with Kaposi’s sarcoma and CD4 nadir of 80 cells/mm^3^; TM decided that ART was indicated. |
|  | Patient with history of extra-pulmonary TB and CD4 nadir of 56 cells/mm^3^; TM decided that ART was indicated. |
|  | New patient, clinically well, no CD4 available yet. TM determined that there was as yet no indication for ART. |
| **Cases in which the TM believed that ART should be continued (without modification) or initiated immediately (during the observed clinical encounter) but the clinical observer disagreed (17.3%).** | |
|  | Robust-appearing patient (weighing 80 kg) with undocumented complaint of weight loss. TM wanted to initiate ART for the indication of “weight loss”. |
|  | Patient with Pott’s disease, abnormal liver function tests, and peripheral neuropathy, on rifampin-based TB treatment. TM wanted to continue 1^st^ line ART, clinical observer recommended switch from nevirapine to efavirenz and from d4T to AZT. |
|  | New patient, no CD4 count, suspicion of pulmonary tuberculosis but not yet evaluated. TM wanted to initiate ART, clinical observer recommended pursuing evaluation of TB first. |
|  | Patient with pancytopenia, markedly abnormal liver and kidney function tests. TM wanted to start 1^st^ line ART. |
|  | Patient with tuberculous lymphadenitis, just starting TB treatment. TM wanted to start ART immediately; clinical observer recommended waiting until patient stable on TB regimen. |
| **Cases in which the TM did not believe that ART was indicated but the clinical observer disagreed (4.7%)** | |
|  | Cachectic patient with chronic, deep soft-tissue infection and suspicion of Pott’s disease; the clinical observer thought that ART was indicated (after evaluation for tuberculosis) but TM did not. |
|  | Patient with Kaposi’s sarcoma, not recognized by TM. |
