# Supplementary material for: Task shifting in Mozambique: cross-sectional evaluation of non-physician clinicians' performance in HIV/AIDS care
Source: Hum Resour Health. 2010 Oct 12;8:23. doi: 10.1186/1478-4491-8-23 (PMC2994547; doi:10.1186/1478-4491-8-23)
Supplement: Additional file 7 — Adverse drug reactions: examples of concordance and disagreement between clinical observers and técnicos de medicina. [file 1478-4491-8-23-S7.DOC]

## Additional file 7 - Adverse drug reactions: examples of concordance and disagreement between clinical observers and *técnicos de medicina*

| **Cases in which the TM and the clinical observer agreed about diagnosis and management of ADR (9/127 [7.1%] of all patients, and 9/78 [11.5%] of patients on CTX prophylaxis, ART, or TB treatment, had adverse drug reactions and observer and *técnico* agreed).** | |
| --- | --- |
|  | Patient with Stevens-Johnson syndrome caused by co-trimoxazole. The TM correctly identified the complication and switched the patient to dapsone. |
| **Cases in which the clinical observer and *técnico* disagreed about ADR diagnosis and/or management (11/127 [11.8%] of all patients, 11/78 [14.1%] of patients on CTX prophylaxis, ART, or TB treatment, had adverse drug reactions and clinical observer and TM disagreed).** | |
|  | Patient recently started on 1^st^ line ART with nausea, vomiting, malaise, and an enlarged tender liver. The clinical observer suspected nevirapine hepatitis. The TM did not suspect ADR. |
|  | Patient on 1^st^ line ART with Stevens-Johnson syndrome, probably caused by nevirapine. The TM recognized the Stevens-Johnson syndrome but did not know that this was a reason to discontinue nevirapine. |
|  | Patient with severe anaemia, on co-trimoxazole. The TM did not realize that the anaemia and the co-trimoxazole prophylaxis could be related. |
|  | Patient with peripheral neuropathy severe enough to interfere with her ability to work, likely caused by d4T, not detected by the TM. |
